# New progress in the role of microRNAs in the diagnosis and prognosis of triple negative breast cancer

**DOI:** 10.3389/fmolb.2023.1162463

**Published:** 2023-04-13

**Authors:** Yeqin Fu, Qiuhui Yang, Hongjian Yang, Xiping Zhang

**Affiliations:** ^1^ Department of Breast Surgery, Zhejiang Cancer Hospital, Hangzhou, Zhejiang, China; ^2^ Wenzhou Medical University, Wenzhou, Zhejiang, China

**Keywords:** triple negative breast cancer, miRNA, diagnosis, prognosis, drug resistance

## Abstract

Triple negative breast cancer is distinguished by its high malignancy, aggressive invasion, rapid progression, easy recurrence, and distant metastases. Additionally, it has a poor prognosis, a high mortality, and is unresponsive to conventional endocrine and targeted therapy, making it a challenging problem for breast cancer treatment and a hotspot for scientific research. Recent research has revealed that certain miRNA can directly or indirectly affect the occurrence, progress and recurrence of TNBC. Their expression levels have a significant impact on TNBC diagnosis, treatment and prognosis. Some miRNAs can serve as biomarkers for TNBC diagnosis and prognosis. This article summarizes the progress of miRNA research in TNBC, discusses their roles in the occurrence, invasion, metastasis, prognosis, and chemotherapy of TNBC, and proposes a treatment strategy for TNBC by interfering with miRNA expression levels.

## 1 Introduction

The International Agency for Research on Cancer (IARC) of the World Health Organization has released its latest global cancer data for 2020, revealing that breast cancer has surpassed lung cancer as the most common cancer in the world for the first time. The survival rate of breast cancer patients has significantly increased due to continuous advancements in early identification, individualized treatment, and chemotherapy approaches. However, it remains the leading cause of cancer-related deaths among women worldwide. Compared with other BC(Breast cancer) subtypes, TNBC(Triple negative breast cancer) has a worse prognosis and a higher early recurrence rate, typically with distant metastasis, owing to the absence of ER (Estrogen receptor),PR (Progesterone receptor) and Her-2(Human epidermal growth factor receptor 2) (Amorim et al., 2016; Volovat et al., 2020). TNBC makes for 10%–20% of all breast cancer cases, and the patients tend to be increasingly young (Shen et al., 2015; Araujo et al., 2022). Patients with metastatic TNBC have a median overall survival of about 18 months, and it is significantly less than patients with PR, ER positive, and HER2 enriched diseases, who may have a survival time of more than 5 years (Vagia et al., 2020). In order to improve the survival rate, it is necessary to identify its predictive biomarkers to assess metastatic rate, therapeutic effect, and even to create novel therapeutic approaches. MicroRNAs (miRNAs) are one of the promising molecular targets.

**FIGURE 1:**
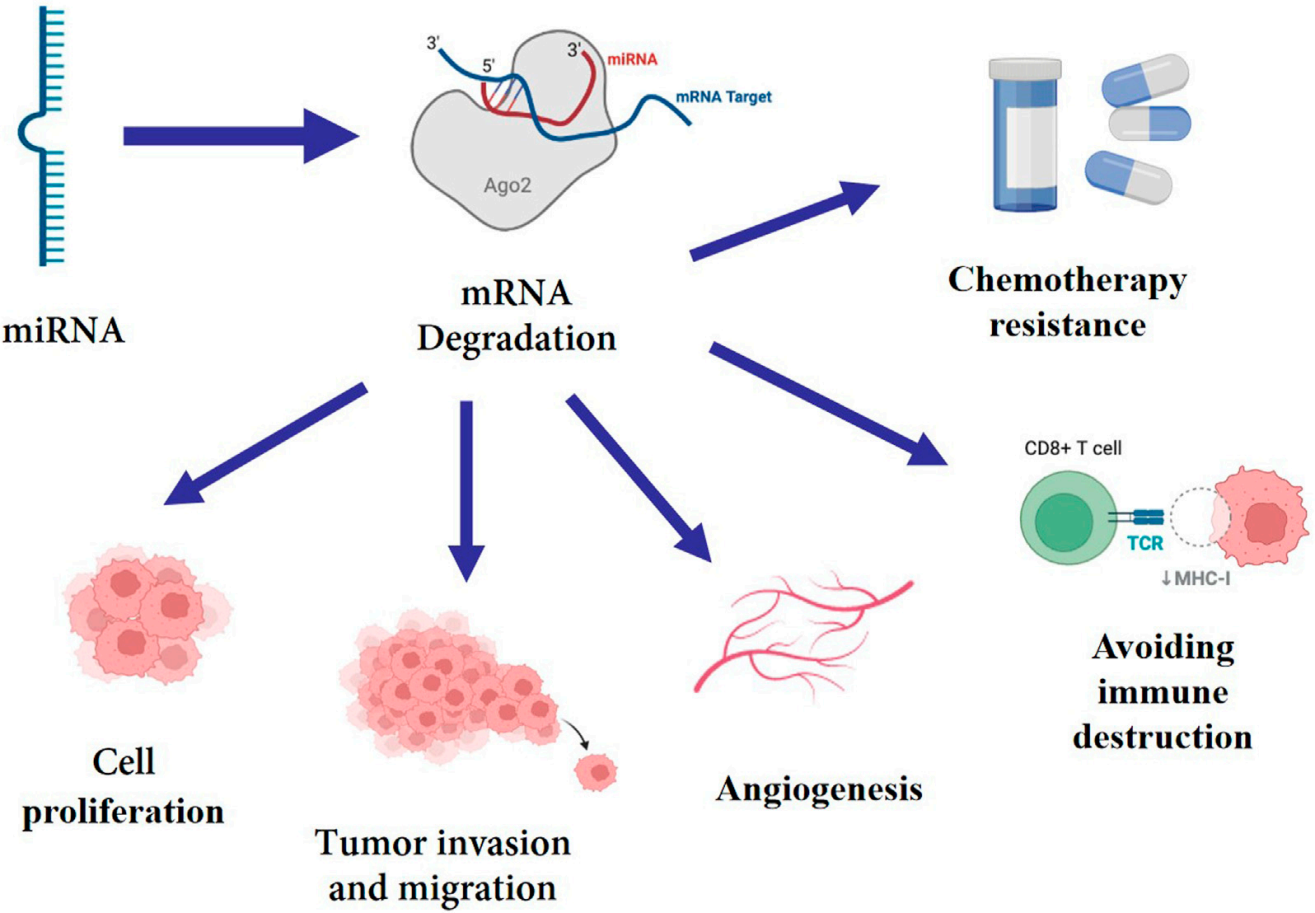
miRNA targets mRNA, mediating its degradation, which enhance cell proliferation, tumor invasion, migration, angiogenesis, immune evasion and chemotherapy resistance.

MiRNAs can be involved in regulating various pathophysiological processes, including proliferation, stress response, cell adhesion, inflammation, as well as cell survival, aging and apoptosis, all of which are closely related to the development of tumors (Xu et al., 2020; Rupaimoole and Slack, 2017). Multiple investigations have demonstrated that miRNA can target certain mRNAs to regulate a number of genes at the pre- and post-transcriptional levels (Du et al., 2020), hence promoting tumor growth, migration, invasion, angiogenesis, immune evasion and chemotherapy resistance (Miyoshi et al., 2017; Qu et al., 2017). (Figure 1) As a consequence, the abnormal expression of miRNA is closely associated with the occurrence and progression of BC. Some miRNAs have been identified to correlate with breast cancer subtypes and can be serve as the potential therapeutic application for BC. In perticular, miR-29a, miR-181a, miR-652 are related to Luminal A subtype (McDermott et al., 2014), miR-342 Luminal B subtype (Lowery et al., 2009), and miR-10b, miR-21 are related with HER-2 positive subtype (Anfossi et al., 2014). Furthermore, miRNA dysregulation contributes significantly to the activation or repression of TNBC-related gene expression. In recent years, numerous studies have discovered that the expression of miRNA in tumor tissue or blood of TNBC patients differs significantly from that of normal people, implying that miRNA may be closely relevant to the formation and progression of TNBC. High-throughput sequencing techniques have been established and developed, which has sped up and improved the accuracy of miRNA identification and expression detection (Thomas et al., 2019). In addition to traditional Northern blot method and fluorescent quantitative PCR technology, the second-generation sequencing (NGS) and microarray technology are being used to detect miRNA expression level (Hamam et al., 2017). Many oncology researchers use TCGA, GEO and other databases to analyze the relationship between miRNA imbalance and tumor occurrence and development (Urabe et al., 2019). This cutting-edge technique for bioinformatics analysis is crucial for the creation of miRNA biomarkers. In this paper, we reviewed recent research findings on miRNA in the diagnosis and prognosis of TNBC, and evaluated the prospects and viability of this field.

## 2 MiRNA with diagnostic and prognostic function

TNBC lacks specific diagnostic and prognostic markers due to tumor heterogeneity (Vargas and Harris, 2016). Currently, the diagnosis of TNBC is primarily based on the pathological detection and immunohistochemical detection, which is time-consuming and costly. It will greatly improve the diagnosis and treatment efficiency of TNBC, if one or several miRNAs can be identified to guide the clinical diagnosis and prognosis of TNBC. The prognosis can be early assessed using TNBC prognostic markers, and improved with early intervention.

### 2.1 miRNA assisting in diagnosis of TNBC

The existence of tumor markers in serum may result from the death of tumor cells, which are released into the blood after splitting, or it is believed to result from the spontaneous secretion of tumor cells. However, the mechanism underlying these two possibilities needs further investigation. Changes in the biological origin of microRNA, epigenetic regulation, transcription factors, and mutant proteins all contribute to altered microRNA expression patterns in breast cancer. It is reported that miRNA-9, miRNA-10b and miRNA-17-5p are abnormally upregulated in TNBC and have the potential to serve as diagnostic markers (Malla et al., 2019). Kahraman et al. used microarrays and Real Time Quantitative PCR(RT-qPCR)to assess the miRNA levels in the blood of healthy women and TNBC patients. They discovered that individuals with TNBC had considerably higher serum concentrations of miR-144-3p, miR-144-5p, miR-126-5p, and let-7d-5p than healthy women. Therefore, by identifying the presence of the aforementioned four miRNAs in patients’ blood, it may be possible to early diagnose TNBC (Kahraman et al., 2018). Using the rank aggregation method to conduct meta-analysis and integrating the miRNA expression profile data set of TNBC, Naorem et al. discovered six kinds of seriously dysregulated miRNAs (miR-135b-5p, miR-18a-5p, miR-9-5p, miR522-3p expression upregulated, miR-190b and miR-449a downregulated) with high prediction accuracy (Naorem et al., 2019). These six miRNAs may be a promising candidate for TNBC diagnostic biomarkers. Yang et al. demonstrated that the level of miR-195-5p in TNBC tissues is lower than that in healthy tissues using TCGA RNA sequence data analysis. Additionally, they measured the levels of miR-195-5p in 40 pairs of TNBC tissues and adjacent non-cancerous tissues, showing that it was considerably downregulated in TNBC tissues compared to paracancerous tissue (Yang et al., 2019). The above miRNAs are expressed differently in TNBC and normal tissues, and it is anticipated that they will function as biomarkers for the diagnosis of TNBC.

As a result of the differentiating expression of several miRNAs between TNBC and other types of BC, TNBC is predicted to be identifiable in many BC patients and these miRNAs will likely serve as biomarkers for differentiating between the various types of BC. Braicu et al. discovered that the miR-17-92 clusters (miR-17, miR-20a, miR-20b, and miR-93), miR-130, miR-22, and miR-29a/c can distinguish TNBC and DPBC (double positive breakthrough cancer, DPBC: ER+, PR+, Her-2) (Braicu et al., 2018). According to Pascull et al., TNBC subgroups had the highest levels of miR-155 expression relative to Luminal A and Luminal B, and HER2 amplification subgroups (Pasculli et al., 2020). Niedwiecki et al. examined the serum miR-200c levels in patients with two different BC subtypes and observed that TNBC patients had lower miRNA-200c levels than the ER/PR positive group as a whole (Niedzwiecki et al., 2018). In addition, there were statistically significant variations of miR-205 expression levels in the BC patients with different ER/PR states. Both the PR positive group and the ER positive group had higher levels of miR-205 than the PR negative group and the ER negative group, respectively. And the expression of miR-205 was found to be considerably higher in the ER+/PR + group compared to the ER -/PR - (i.e., TNBC) group. As a result, miR-205s low expression has an indication for TNBC (Petrovic et al., 2022). (Table 1)

**TABLE 1:** Diagnosis related MicroRNAs.

microRNA	Type of deregulation in TNBC	Samples type	References
Differentially expressed miRNAs between TNBC and Non-TNBC			
miRNA-9,miRNA-10b,miRNA-17-5p	Upregulated	Tumor Tissues	Malla et al. (2019)
miR-144-3p, miR-144-5p, miR-126-5p and let-7d-5p	Upregulated	Serum	Kahraman et al. (2018)
miR-135b-5p,miR-18a-5p, miR-9-5p, miR-522-3p	Upregulated	Tumor Tissues	Naorem et al. (2019)
miR-190b,miR-449a	Downregulated	Tumor Tissues	Naorem et al. (2019)
miR-195-5p	Downregulated	Tumor Tissues	Yang et al. (2019)
**Differentially Expressed miRNAs Between TNBC and other breast cancer (BC) Subtypes**			
miR-17–92 (miR-17,miR-20a,miR-20b and miR93)and miR-130,miR-22, miR-29a/c	Upregulated	Tumor Tissues	Braicu et al. (2018)
miR-155-5p	Upregulated	Tumor Tissues	Pasculli et al. (2020)
miR-200c	Downregulated	Tumor Tissues	Niedzwiecki et al. (2018)
miR-205	Downregulated	Tumor Tissues	Petrovic et al. (2022)

Some biomedical companies and universities have developed specific miRNA detection kits for BC diagnosis in recent years, but there has been no report on targeted miRNA detection kits for TNBC diagnosis. We believe that it will be available soon.

### 2.2 miRNA assisting in determining prognosis

It will be absolutely crucial for the treatment of TNBC if we can predict the relationship between the up/downregulation of particular miRNAs and the prognosis of TNBC in order to determine the overall survival (OS), disease-free survival (DFS), and distant metastasis free survival (DMFS) of TNBC patients. We speculate that miRNA can be used as an effective therapeutic strategy and prognostic marker for TNBC. Weng et al. considered that the expression of the oncogene Multiple Copies in T-cell Malignancy 1 (MCT-1/MCTS1), which functions through MCT-1/miR-34a/IL-6/IL-6R, is a novel poor prognostic sign for patients with TNBC. By preventing the expression of IL-6R, which is supported by MCT-1, MiR-34a can enhance the prognosis of TNBC (Tsiakou et al., 2019; Weng et al., 2019). As a result, boosting miR-34a expression aids in improving TNBC’s prognosis. MiR-374a-5p can target arrestin beta 1 (ARRB1) and reduce its expression. Additionally, the expression of ARRB1 is positively connected with the survival rate of TNBC patients and negatively correlated with the histological grade of breast cancer (Son et al., 2019). Tormo et al. revealed that miR-449a high expression was noticeably associated with favourable prognosis, whereas miR-449b/c was unrelated to prognosis, based on their analysis of the GEO database (Tormo et al., 2019). Using tissue microarray (TMA), Yao et al. investigated the expression of miR-493 in breast cancer samples and found that patients with high miR-493 expression had improved DFS (Zhao et al., 2016a; Yao et al., 2018). Through bioinformatics analysis, Qiu et al. noticed that miR-3163 was connected to the poor OS of androgen receptor (AR) positive TNBCs. These results indicate that miR-3163 may be promising prognostic markers and therapeutic targets for AR positive TNBCs (Qiu et al., 2021). Through meta-analysis, Qattan et al. demonstrated an association of upregulated miR-93 and miR-210 with poor OS outcomes in TNBC patients (Li et al., 2017a; Du et al., 2020; Qattan et al., 2021). Hsiao Chin Hong et al. mined TCGA and GEO databases by logistic regression analysis and Gaussian mixture model, and then used the Kaplan-Meier method to conduct a comprehensive survival analysis, revealing that miR-455-3p was significantly related to OS, while miR-139-5p was significantly related to DFS, indicating that them were related to the recurrence of TNBC (Li et al., 2017b; Hong et al., 2020). All of the above investigations demonstrated that some particular miRNAs were associated with patient survival, prognosis, and recurrence and were therefore considered to be potential prognostic markers of TNBC (Table 2).

**TABLE 2:** Abnormal expression of MicroRNAs that can judge prognosis.

MicroRNA	Type of DeregulationIn TNBC	Mechanism	Biological function	Referance
miR-34a	Upregulated	MCT-1/miR-34a/IL-6/IL-6R	Poor OS	Weng et al., (2019), Tsiakou et al., (2019)
miR-374a-5p	Upregulated	Targeting ARRB1	Good OS and DMFS	Son et al. (2019)
miR-449a	Upregulated	Sensitized cells to the treatment and reduced theresistance to doxorubicin	Good OS	Tormo et al. (2019)
miR-493	Upregulated	Targeting of fucosyltransfer-ase IV	Good DFS	Yao et al., 2018, Zhao et al., (2016a)
miR-3163	Downregulated	Targeting CCNB1	Poor OS	Qiu et al. (2021)
miR-93	Upregulated	SFPR1/Wnt/β-catenin	Poor OS	Qattan et al., (2021), Li et al., (2017a)
miR-210	Upregulated	Targeting GPD1L to maintainHIF-1α stabilization and CYGB to suppress p53	Poor OS	Qattan et al., (2021), Du et al., 2020
miR-445-3p	Upregulated	Targeting EI24	Poor OS	Hong et al., 2020, Li et al., 2017b
miR-139-5p	Downregulated	Targeting ARF6	Poor DFS	Hong et al. (2020)

In conclusion, miRNA imbalance may become a potentially important tool for identifying key biomarkers in patients with TNBC. The above indicators can be detected by the patient’s blood or tumor tissue, with high sensitivity and specificity. In this case, miRNA not only can be employed as a potential marker to distinguish TNBC from other breast cancer, but also can be used as a biomarker, participating in canceration, predicting prognosis and evaluating treatment response. It is anticipated to be used in conjunction with conventional invasive biopsy to diagnose TNBC and predict its prognosis.

## 3 MiRNAs promoting tumor formation and progression

Tumor academia has come to terms with the notion that miRNA has a role in the initiation and progression of breast cancer, particularly TNBC, a heterogeneous subtype of the disease. Breast cancer progression is directly correlated with tumor growth, invasion, migration, and angiogenesis. Currently, it has been proven that a number of miRNAs participate in the aforementioned physiological processes of TNBC tumor cells, which can encourage the formation and progression of tumors. As a result, miRNA-based research is crucial for the early diagnosis and management of TNBC.

**FIGURE 2:**
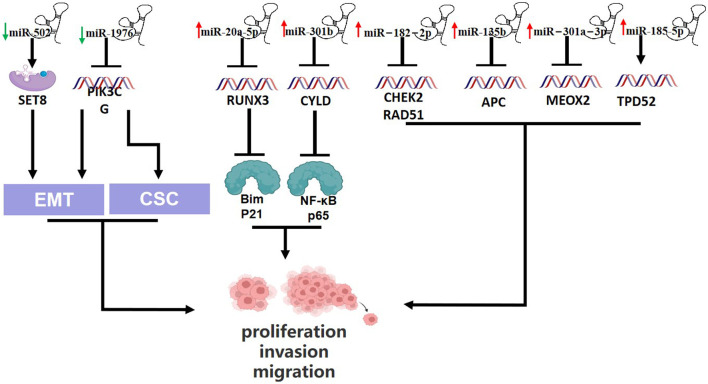
Schematic presentation of miRNAs involvement in promotion of triple-negative breast cancer and relationship between miRNAs and epithelial-to mesenchymal transition (EMT) and Cancer stem cells-like properties (CSC). Downregulated miR-502 enhance SET8 expression, which promotes EMT. miR-1976 knockdown could enhance EMT and CSCs by blocking PIK3CG. Upregulation of miR-20a-5p suppresses RUNX3, which suppresses Bim and p21. miR-301b is upregulated and represses CYLD, which block NF-KB p65. And high expression of miR-182-2p suppresses CHEK2 and RAD51. Upregulated miR-135b, miR-301a-3p and miR-185-5p block APC, MEOX2 and TPD52, respectively.

miR-20a-5p promotes TNBC cell proliferation by targeting Run related transcription factor 3 (RUNX3) and its immediate downstream targets Bim and p21 (Bai et al., 2018). miR-135b is a highly expressed miRNA in TNBC that targets the 3′-UTR of APC, which helps tumor cells proliferate and metastasize (Lv et al., 2019). A study in 2021 found that MDA-MB-231 cells (TNBC cells) expressed more miR-301a-3p than MCF-10A cells (human breast epithelial cells) did. Furthermore, miR-301a-3p overexpression can suppress mesenchymal homeobox 2 (MEOX2) expression, increasing the viability, migration and invasion of MDA - MB - 231 cells (Liu and Wang, 2021). In addition to targeting the appropriate genes to control the development of TNBC, some miRNAs can promote tumor progression by controlling the cell cycle and thwarting tumor cell apoptosis. And miR-502 could directly target H4K20 methyltransferase SET8, which is involved in cell proliferation and cycle, to encourage the transition of the cell cycle from the G phase to the M phase (Liu et al., 2016; Cantini et al., 2019). In TNBC tumor tissue, miR-301b was shown to be upregulated, and Song et al. discovered that it directly bound to the 3′-UTR of the CYLD lysine 63 diquinase mRNA to activate NF-kB p65 and prevent 5-FU from inducing tumor cell apoptosis (Song et al., 2018). Carcinogenic miRNA is linked to promoting the growth and invasion of TNBC tumor cells as well as tumor metastasis. Recently, Darbeheshti et al. discovered that miR-182-5p was considerably upregulated in TNBC tumor tissues compared to adjacent normal tissues, and that high miR-182-5p expression has a strong correlation with larger tumors, higher tumor grades, and positive lymph nodes. Moreover, miR-182-5p overexpression accelerates TNBC development and lymph node metastasis by downregulating the genes CHEK2 and RAD51 (Darbeheshti et al., 2022). In some way, the progress of TNBC is maintained by recruiting powerful tumor microenvironments (TMEs), which are mainly composed of cancer related fibroblasts (CAFs) that can recognize tumor markers. Scognamiglio et al. dicovered that the synergistic action of miR-185-5p, miR-652-5p and miR-1246 promoting fibroblast migration, and specific cancer-associated fibroblasts towards a pro-migratory functional state, finally boosting TNBC progression and migration (Scognamiglio et al., 2022). Wang et al. revealed that miR-1976 knockdown could enhance EMT and CSCs *in vitro* by targeting PIK3CG (Phosphatidylinositol-4,5-bisphosphate 3-kinase catalytic subunit gamma) (Wang et al., 2020). (Figure 2)

The abnormal expression of the above-mentioned miRNAs in TNBC promotes tumor cell formation, proliferation, invasion, and metastasis by targeting specific genes, regulating the cell cycle, inhibiting tumor cell apoptosis, and other mechanisms. These mechanisms also provide us with new therapeutic strategies. Whether we can use inhibitors of these molecules or block relevant signal pathways to control TNBC tumor cell proliferation and invasion speed, or even cause them to die due to a lack of relevant growth factors. A significant number of miRNAs that are abnormally expressed in TNBC are also listed in the TCGA and other tumor datasets in addition to the miRNAs mentioned above. These miRNAs can be employed as cancer-causing genes to take part in EMT, CSCs maintenance, epigenetic alterations, and other processes. Experiments are necessary to further our demonstration.

## 4 MiRNA inhibiting tumor formation and progression

Despite prior research on the role of miRNA in the initiation and progression of cancers, the role of miRNA in TNBC is not limited to this. It has been reported that some miRNAs can inhibit tumor formation, proliferation, invasion and migration, thereby preventing TNBC progression.

### 4.1 miRNA inhibiting tumor formation, proliferation and invasion

MiRNA-29c prevents preneoplastic TNBC cells from proliferating and populating the body by directly interacting with and regulating TGFB-induced factor homeobox 2 (TGIF2), CAMP-responsive element binding protein 5 (CREB5), and V-Akt murine thymoma viral oncogene homolog 3 (AKT3). As a result, miRNA-29c exerts significant influence during the early, preneoplastic phases of TNBC development (Bhardwaj et al., 2017). Wu et al. found that the downregulation of miR182-5p inhibits the production of inflammatory factors and the activation of inflammatory signals in TNBC cells by targeting FBXW7, thereby inhibiting the proliferation and invasion of TNBC and promoting apoptosis (Wu et al., 2020). Highly expressed in human TNBC tissues and cell lines, miR-125b inhibits proliferation and metastasis by binding 3′- UTR of APC, and preventing Wnt/β-catenin signaling pathway and EMT in cells (Nie et al., 2019). Lee et al. discovered that miR-496, which targets Del-1, prevents TNBC cancer cells from proliferating (Lee et al., 2021). MiR-890 negatively regulates the target gene CD147 in TNBC cells, which suppresses cell proliferation and invasion and induces apoptosis (Wang et al., 2019a). By interacting with the 3′-UTR of EZH2 mRNA, miR-1301 inhibits the proliferation, migration, and colony formation of TNBC cells after overexpression both *in vivo* and *in vitro* (Wu et al., 2018).

The above miRNAs inhibit the proliferation and invasion of TNBC by targeting specific genes. It is hoped that more mechanisms can be found in the future to inhibit the progress of TNBC.

### 4.2 miRNA inhibiting tumor metastasis

The capacity of primary breast cancers to metastasize is a essential aspect when grading and staging tumors and determining therapeutic approaches based on their clinical characteristics. Tumor suppressor miRNAs are vital for preventing the invasion, metastasis, and migration of tumor cells. Ismael et al. believed that overexpression of miR-149 in MDA-MB-231 cells inhibited THP-1 macrophage recruitment. By targeting CSF1, miR-149 inhibits CSF1 dependent communication between TNBC cells and THP-1 macrophages, thereby blocking the paracrine interactions between MDA-MB-231 cells and THP-1 cells and inhibiting breast cancer metastasis (Sanchez-Gonzalez et al., 2020). By MTT, colony formation, Transwell, xenotransplantation models in naked mice, Zhang et al. revealed that miR-574-5p levels were decreased in breast cancer tissues and cells, inhibited the proliferation, migration, and EMT of TNBC cells, and decreased the size and metastasis rate of tumors *in vivo* (Zhang et al., 2020). Additionally, some miRNAs target the corresponding targets to prevent local lymph node metastasis and/or distant metastasis of TNBC. Examples include miR-33b targeting HMGA2, SALL4, and Twist1 (Lin et al., 2015), miR-124 targeting ZEB2 (Ji et al., 2019), miR-126-3p targeting regulator of G protein signal 3 (RGS3) (Hong et al., 2019), miR-130a targeting FOSL1 and zona occludens 1 (zonula clusters 1, ZO-1 or called TJP1) (Chen et al., 2018), miR-145 targeting ARF6 (ADP-ribosylation factor 6) (Eades et al., 2015).

Some miRNAs can prevent TNBC from progressing by blocking the proliferation and peripheral infiltration of tumor cells as well as local and distant metastasis. The first discovered epigenetic regulated miRNA, miR-127, has been shown to target the gene BL6 (Saito et al., 2006) and suppress its expression in breast cancer (Chen et al., 2013; Wang et al., 2014). Garcia et al. confirmed that miR-127PD drastically decreased the activity of TNBC cells. And they observed a reduction in lung metastasis in mice treated with the miR-127PD system (Umeh-Garcia et al., 2020). miR-127PD also significantly reduced the spherulation capacity of four TNBC cells (MDA MB-231, MDA MB-157, MDA MB-468, HCC 1937), which was more effective than miR-34a (Raver-Shapira et al., 2007; Zhao et al., 2015; Adams et al., 2016; Zhao et al., 2016b). While miR-127PD is the precursor of miR-127-3p, which accumulates in cells after being processed and matured. Therefore, it makes sense to assume that miR-127-3p can prevent TNBC tumor cells from proliferating. They further used the tumorsphere assays, a widely accepted stem cell function test, and confirmed that miR-127 inhibited CSC, which has been of great significance in preventing the metastasis of TNBC and disease recurrence (Umeh-Garcia et al., 2020). (Table 3)

**TABLE 3:** MicroRNAs inhibit TNBC.

MicroRNA	Change in TNBC	Mechanism	Biological function	References
Inhibit proliferation and invasion				
miRNA-29c	Downregulated	Targeting TGIF2, CREB5 and AKT3	Inhibit proliferation	Bhardwaj et al. (2017)
miR-182-5p	Downregulated	Targeting FBXW7 to regulate TLR4/NF-κB pathway	Inhibit proliferation and invasion	Wu et al. (2020)
miR-125b	Upregulated	Targeting APC to regulate Wnt/β-cateninpathway	Inhibit proliferation and invasion	Nie et al. (2019)
miR-496	Downregulated	Targeting Del-1	Inhibit proliferation and invasion	Lee et al. (2021)
miR-890	Downregulated	Targeting CD147	Inhibit proliferation and invasion Induces apoptosis	Wang et al. (2019a)
miR-1301	Upregulated	Targeting EZH2	Inhibit proliferation, invasion and colony formation	Wu et al. (2018)
inhibit migration				
miR-149	Upregulated	Targeting CSF1	Inhibit metastasis	Sanchez-Gonzalez et al. (2020)
miR-574-5p	Downregulated	Targeting SOX2 and BCL11A	Inhibit proliferation, invasion and EMT	Zhang et al. (2020)
miR-33b	Downregulated	Targeting HMGA2, SALL4 and Twist1	Inhibit metastasis and CSC	Lin et al. (2015)
miR-124	Downregulated	Targeting ZEB2	Inhibit EMT and metastasis	Ji et al. (2019)
miR-126-3p	Downregulated	Targeting RGS3	Inhibit proliferation, migration, invasion, colony formation capacity and angiogenesis	Hong et al. (2019)
miR-130a	Upregulated	Targeting FOSL1 and ZO-1	Inhibit metastasis and invasion	Chen et al. (2018)
miR-145	Downregulated	Targeting ARF6	Inhibit metastasis and invasion	Eades et al. (2015)

Metastasis is the most serious complication and leading cause of death in cancer patients. At present, breast cancer patients with distant metastasis essentially no longer have access to surgical treatment, and the median survival time is measured in months. TNBC has a greater capacity for invasion and metastasis than other BC types. If the key miRNAs that can promote the invasion and metastasis of TNBC are identified and blocked, the local metastasis of tumor cells, the ability of distant metastasis, and the mortality can be reduced, as well as the chance of radical surgery and the survival period can be increased. TNBC patients can benefit significantly from this.

## 5 Relationship between MicroRNA and chemotherapy resistance of tumor

Chemotherapy resistance is a major hindrance to neoadjuvant therapy. Unfortunately, treatment resistance is highly common, and this is one of the leading factors contributing to TNBC patients’ poor prognosis. MiRNA expression disorders, such as the upregulation of carcinogenic miRNA and the downregulation of tumor suppressor miRNA, are frequently seen in chemotherapy-resistant cancer cells. The uncontrolled expression of miRNA can be extrapolated into a direct connection to TNBC’s treatment resistance. It is now widely acknowledged that doxorubicin and platinum resistance are related to aberrant miRNA expression. Other chemoresistance research with miRNA are increasingly conducted as well.

### 5.1 miRNAs associated with doxorubicin and platinum chemoresistance

It is reported that the downregulation of miR-129-5p makes it resistant to doxorubicin (DOX or adriamycin) by promoting the apoptosis resistance induced by Sex-Determining Region Y-Box 2 (SOX2) (Zeng et al., 2018). By downregulating the multidrug resistance gene 1 (MDR1) and decreasing DOX efflux, increasing miR-145 expression can boost the sensitivity of MDA-MB-231 cells to DOX both *in vitro* and *in vivo* (Wang et al., 2021). While miR-154 promotes nicotinamide phosphoribosyltransferase (NAMPT), which further increases DOX chemical resistance, to prevent cell death (Bolandghamat Pour et al., 2019). Adenosine triphosphate binding cassette (ABC) transporter overexpression is the primary mechanism of acquired drug-resistance in multidrug-resistant cancer (Robey et al., 2018). While the increase of ABCC3(ATP binding cassette transporter family class C3) is related to the reduction of miR-181b-2-3p in MDA-MB-231/DOX (MDA-MB-231 cells resistant to DOX), decreasing the sensitivity of TNBC cells to DOX (Zeng et al., 2020). Furthermore, some miRNAs, such as miR-26a-5p, miR-142-3p, miR-200, and miRNA-5195-3p, are correlated with PTX resistance. Upregulation of miR-26a-5p promoted cellular cytotoxicity of PTX *in vitro* and *in vivo* (Li et al., 2021a). Higher miR-142-3p expression increased sensitivity to PTX treatment (Shi et al., 2023). MiRNA-200 inhibited PTX resistance (Li et al., 2018). PTX-resistant TNBC cells responded better to PTX therapy when miR-5195-3p was upregulated (Liu et al., 2019).

Platinum based first-line treatment are typically effective against breast cancer, and they can increase patients’ DFS, PFS, and ORR in both the early and late stages of TNBC. However, many patients with TNBC experience recurrence due to drug resistance, which lessens the therapeutic impact of cisplatin (DDP) on TNBC. MiR-105/93-3p is overexpressed in TBNC, which activate Wnt/β-catenin transmits signals by downregulation of SFRP1, thus endowing TNBC cells with cisplatin resistance (Li et al., 2017a). MiR-145-5p in MDA-MB-231 cells induces apoptosis and increases sensitivity to cisplatin therapy by downregulating transforming growth factor TGFβR2, albeit the precise regulatory mechanism is still unknown (Garcia-Garcia et al., 2019). And, the upregulation of miR-423-5p contributed to the drug resistance of MDA-MB-231 cells and had a substantial impact on the DDP resistance (Wang et al., 2019b).

Through pertinent pathways, these miRNAs contribute to doxorubicin and platinum resistance in TNBC. It is hoped that drug resistance can be overcome in the future to maximize the anti-tumor efficacy of doxorubicin and platinum.

### 5.2 MiRNAs that cause chemoresistance through other mechanisms

MiRNAs can directly target associated proteins or further regulate related signal pathways to regulate chemotherapy resistance. According to Cheng et al., chemotherapy-resistant cells have significantly higher levels of FSTL1, which was necessary for DDP and DOX chemoresistance in breast cancer cell lines. And there was a miR-137/FSTL1/integrinβ3/Wnt/β- Catenin signal axis maintaining stemness and enhancing chemoresistance in breast cancer cells (Cheng et al., 2019). Saatci et al. believed that inhibiting lysyl oxidase (LOX) can decrease collagen cross-linking and fibronectin assembly, boost drug absorption, and downregulate the expression of ITGA5/FN1, which inhibits FAK/Src signal transduction, induces apoptosis, and boosts chemotherapy sensitivity. Upregulation of miR-142-3p expression can target HIF-1α/The LOX/ITGA5/FN1 axis further inhibits the chemoresistance of TNBC (Saatci et al., 2020). As a tumor inhibitor, miR-17 inhibits the resistance of TNBC to DDP by promoting the expression of cell cycle inhibitor p27 and apoptosis. Therefore, miR-17, as a tumor chemotherapy sensitizer, may be an effective biological target to combat TNBC resistance (Wang et al., 2019c).

Additionally, a variety of mechanisms, including altered cell cycle and DNA damage regulation, decreased drug absorption, increased drug excretion, and others, might affect cancer drug resistance (Najminejad et al., 2019; Si et al., 2019). MiRNAs, as tissue specific regulators of the entire gene network related to drug resistance, have become research frontiers. Qattan et al. found that with had significantly aberrant expression of miR-19a/b-3p, miR-25-3p, miR-22-3p, miR-210-3p, miR-93-5p, and miR-199a-3p in TNBC patients is relavent to chemotherapy resistance. These miRNAs control the PAM (PI3K/Akt/mTOR), HIF-1, TNF, FoxO, Wnt and JAK/STAT, PD-1/PD-L1 pathway and EGFR tyrosine kinase inhibitor resistance (TKI) respectively (Qattan et al., 2021). In addition to miRNAs involved in chemotherapy resistance through the above mechanisms, some miRNAs can function as oncogenes. Their upregulation promotes chemotherapy resistance by inhibiting TNBC cell apoptosis, such as miR200a (Yu et al., 2018), miR-221/222 (Li et al., 2019), and miR1207-5p (Hou et al., 2019).

These indicators are especially well suited for addressing the treatment resistance issue of this clinically challenging subtype of breast cancer due to the mounting evidence of miRNA imbalance in TNBC. There is increasing number of studies on miRNA in chemotherapy resistance. We can consider using appropriate miRNA inhibitors or mimics to reverse the resistance to conventional chemotherapy drugs and enhance the effect of chemotherapy drugs. At present, this article focuses on the relationship between miRNA abnormal expression and DOX and platinum resistant TNBC. The connection between miRNA and chemoresistance to drugs like paclitaxel, cyclophosphamide, and capecitabine will then receive additional focus (Mini et al., 2006; Saif, 2009; Li et al., 2018; Zeng et al., 2018; Bolandghamat Pour et al., 2019; Iqubal et al., 2019; Liu et al., 2019; Martins-Teixeira and Carvalho, 2020; Zeng et al., 2020; Li et al., 2021a; Wang et al., 2021; Shi et al., 2023). In conclusion, many miRNAs’ drug resistance mechanisms have not yet been completely grasped, necessitating more research (Table 4).

**TABLE 4:** Common chemotherapeutic drugs for TNBC.

Chemotherapy drugs	Mechanism	Common drugs	MiRNAs associated with drug resistance	References
Anthracyclines	Acting via DNA insertion, oxidative stress production, and topoisomerase II poisoning	Doxorubicin	miR-129-5p,miR-145	Zeng et al., 2018, Wang et al., 2021, Bolandghamat Pour et al., (2019), Robey et al., 2018, Zeng et al., 2020, Martins-Teixeira and Carvalho, (2020)
		Epidoxorubicin	miR-154,miR-181b-2-3p	
Paclitaxel	Promoting intracellular tubulin polymerization and stabilizes abnormal microtubule structures against depolymerization	Docetaxel	miR-26a-5p, miR-142-3p,miR-200,miRNA-5195-3p	Li et al., 2021a, Shi et al., 2023, Li et al., 2018, Liu et al., (2019)
		Nab-paclitaxel		
Platinum	Cross-linking with bases on the DNA chain, damaging the structure and function of DNA	Carboplatin	miR-105/93-3p,miR-145-5p,miR-423-5p	Li et al., 2017a, Garcia-Garcia et al., (2019), Wang et al., 2019b
		Cisplatin		
		Oxaliplatin		
Cyclophosphamide	Undergoing hepatic metabolism and producting aldophosphamide, which decomposes into phosphoramide mustard and acrolein in tumor cells to act cytotoxic effects			Iqubal et al. (2019)
Capecitabine	Entering the body and converting into 5-FU, which is incorporated into RNA in a competitive inhibition manner to interfere with protein synthesis			Saif (2009)
Gemcitabine	Its main metabolite incorporated into DNA within the cell and mainly acting on the G1/S phase, and inhibiting nucleotide reductase, leading to a decrease in intracellular deoxyribonucleotide triphosphate, and inhibiting deoxycytidine deaminase to reduce the degradation of intracellular metabolites			Mini et al. (2006)

## 6 miRNA and precision oncology

Precision oncology seeks to identify individual differences based on the personal genetic information of cancer patients, comprehend the phenotypes of disease, and direct personalized treatment (Yu and Snyder, 2016). Genomics provides valuable information on driving mutations and risk loci, while transcriptomics describes multiple expression patterns of mRNA and non-coding RNA (ncRNA), which can aid in deciphering genomic codes (Ma et al., 2018). The abnormal expression of miRNA in TNBC is also within the scope of precision oncology study because it is an indispensable component of ncRNA in the genome. By analyzing data from The Cancer Genome Atlas (TCGA), numerous scholars have currently examined the imbalance of miRNA in TNBC (Yang et al., 2019; Hong et al., 2020). Thanks to advancements in RNA sequencing, microarray technology, and high-throughput sequencing technology, more miRNA anomalies in TNBC have also been revealed.

In the previous section, we have described the abnormal expression of many miRNAs in TNBC. Upregulated oncogenic miRNAs may act as tumor promoters to increase the proliferation and/or invasion of TNBC cells, whereas downregulated tumor suppressor miRNAs may serve as tumor inhibitors to inhibit cancer cell growth, induce apoptosis, and enhance metastasis. Antisense oligonucleotides, miR mimics, and chemical modification of miRs are examples of current miRANA-based treatments (Nagini, 2017). Through the aforementioned techniques, it might be feasible to downregulate the expression of oncogenic miRNAs and upregulate the expression of tumor suppressor miRNAs, to inhibit the proliferation and invasion of TNBC. Many investigtions have demonstrated that miRNA can target related genes and obstruct their protein translation (Bai et al., 2018; Cantini et al., 2019; Lv et al., 2019; Son et al., 2019) (Wang et al., 2019a; Wu et al., 2020; Lee et al., 2021), which is connected to regulating tumor progression, chemotherapy resistance, and tumor immune surveillance. In the event that modifying the levels of miRNA prove to be challenging, we propose a hypothesis that corresponding antibodies can be created to attach to carcinogenic proteins and render them inactive, thereby slowing tumor progression. Furthermore, based on the abundance and pattern of miRNAs expression, BC subtypes could be reclassified, and the principal roles of related miRNAs were used to determine the precise biological functions of malignancies. Especially TNBC, the tumor is extremely heterogeneous, with ambiguous characteristics, and at this time there is no targeted medication. Therefore, precise target research orientig miRNA to provide individualized treatment for patients will be a blessing for all patients with TNBC.

## 7 Discussion

Breast cancer, the most frequent malignant tumor in women, has put women’s lives in danger worldwide. TNBC, lacking ER, PR and HER-2 expression, has a poor prognosis and is more prone to relapse due to the inability to use endocrine and monoclonal antibody targeted therapy. Epigenetic control, transcription factors, and/or mutant protein control alterations all contribute to altered miRNA expression patterns in breast cancer. Therefore, persistent aberrant miRNA expression may result in the development of tumors. This paper focuses on the role of particular miRNAs in the occurrence, development, and recurrence of TNBC. It also aims to identify some biomarkers that may reliably diagnose TNBC and assess the prognosis from a large pool of miRNAs in the future, so as to improve the prognosis of patients.

Despite numerous research on miRNA, there are currently a few precise, repeatable biological targets for the treatment of TNBC. Some studies have confirmed that miRNA is involved in regulating various metabolic pathways in cells. From the perspective of metabonomics, we can investigate whether miRNAs in TNBC modulate particular metabolic pathways of TNBC in the future, and then discover novel targeted biomarkers. The therapeutic scheme targeting miRNA may bring hope for the treatment of TNBC. A certain miRNA’s up- or downregulation, however, may affect the epigenetics of other tissues and cells, or potentially cause malignant changes, as many miRNAs do not exclusively target breast cells. As a result, there may be some application concerns with miRNA-targeted therapy for TNBC. It is worth considering whether changing the expression of miRNA will cause secondary tumors and damage other parts of the human body, and we must tread cautiously.

Along with miRNA, ohter RNAs, including LncRNA and CircRNA in ncRNA, have also been dicovered to be variably expressed in TNBC. And mounting evidence indicates that they may develop into potential biomarkers for diagnosis and prognosis, therapeutic targets, and improve the clinical outcomes for TNBC patients. For example, elevated expression of lncRNA H19 can be used to predict the efficacy of neoadjuvant chemotherapy (NAC) (Ozgur et al., 2020). NRAD1 is enriched in TNBC populations and is associated with a poor survival rate (Vidovic et al., 2020). And CircSEPT9 (Zheng et al., 2020), CircCD44 (Li et al., 2021b), *etc.*, can be used as indicators of TNBC. It is interesting to note that lncRNA and circRNA can associate with miRNA, which affects how TNBC occurs and develops. The competitive endogenous RNA (ceRNA) theory (Salmena et al., 2011) postulates that lncRNA may function as a “sponge” for miRNA, competing with miRNA-targeted mRNA and influencing miRNA-mediated gene regulation (Poliseno et al., 2010; Cesana et al., 2011). CircRNAs can also regulate the proliferation, invasion and tumorigenesis of TNBC positively or negatively through the circRNA-miRNA-mRNA axis (Tian et al., 2021). This provides a theoretical foundation for our upcoming research on non-coding RNA medications used in combination to treat TNBC.

With a thorough study of TNBC, it is discovered that TNBC can be classified into various subtypes based on different traits and analytical techniques. In the future, we can further study the relationship between miRNAs and different TNBC subtypes, clarify that specific miRNAs play a role in promoting or inhibiting tumor progression in TNBC subtypes, and carry out precise individualized treatment to improve the efficacy. Although the impact of abnormally expressed miRNAs on the development of TNBC has been extensively investigated and the mechanism of some miRNAs has also been revealed. However, therapeutic application of exogenous miRNAs for the treatment of TNBC is rare due to their instability and low specificity *in vivo*. Consequently, it is vital to find solutions to the challenges of miRNA medication stability optimization and miRNA delivery improvement. The addition of miRNA degradation inhibitors can reduce miRNA decomposition and improve the drug stability of miRNA, thereby improving the efficacy. Secondly, medication delivery effects can be maximized and gene targets can be more precisely combined with the aid of nanotechnology or the employment of more than two carriers in a synergistic manner. This review’s key goals are to advance effective, less invasive treatment options, raise awareness of the role of miRNA in the emergence of TNBC, and enhance the prognosis for TNBC as much as feasible.
