# Supplementary material for: New progress in the role of microRNAs in the diagnosis and prognosis of triple negative breast cancer
Source: Front Mol Biosci. 2023 Apr 13;10:1162463. doi: 10.3389/fmolb.2023.1162463 (PMC10134903; doi:10.3389/fmolb.2023.1162463)
Supplement: Supplementary file 1 [file Table1.DOCX]

**Table1: Diagnosis related MicroRNAs**

| **microRNA** | **Type of Deregulation In TNBC** | **Samples Type** | **Reference** |
| --- | --- | --- | --- |
| **Differentially Expressed miRNAs Between TNBC and Non-TNBC** | | | |
| miRNA-9，miRNA-10b，miRNA-17-5p | Upregulated | Tumor Tissues | [18] |
| miR-144-3p, miR-144-5p, miR-126-5p and let-7d-5p | Upregulated | Serum | [19] |
| miR-135b-5p，miR-18a-5p， miR-9-5p， miR-522-3p | Upregulated | Tumor Tissues | [20] |
| miR-190b，miR-449a | Downregulated | Tumor Tissues | [20] |
| miR-195-5p | Downregulated | Tumor Tissues | [21] |
| **Differentially Expressed miRNAs Between TNBC and other breast cancer (BC) Subtypes** | | | |
| miR-17-92（miR-17，miR-20a，miR-20b and miR93）and miR-130，miR-22, miR-29a/c | Upregulated | Tumor Tissues | [22] |
| miR-155-5p | Upregulated | Tumor Tissues | [23] |
| miR-200c | Downregulated | Tumor Tissues | [24] |
| miR-205 | Downregulated | Tumor Tissues | [25] |

**Table 2:** **Abnormal expression of MicroRNAs that can judge prognosis**

| **MicroRNA** | **Type of DeregulationIn TNBC** | **Mechanism** | **Biological function** | **Referance** |
| --- | --- | --- | --- | --- |
| miR-34a | Upregulated | MCT-1/miR-34a/IL-6/IL-6R | Poor OS | [26][27] |
| miR-374a-5p | Upregulated | Targeting ARRB1 | Good OS and DMFS | [28] |
| miR-449a | Upregulated | sensitized cells to the treatment and reduced theresistance to doxorubicin | Good OS | [29] |
| miR-493 | Upregulated | targeting of fucosyltransfer-ase IV. | Good DFS | [30] [31] |
| miR-3163 | Downregulated | Targeting CCNB1 | Poor OS | [32] |
| miR-93 | Upregulated | SFPR1/Wnt/β-catenin | Poor OS | [33][34] |
| miR-210 | Upregulated | Targeting GPD1L to maintainHIF-1α stabilization and CYGB to suppress p53 | Poor OS | [33][8] |
| miR-445-3p | Upregulated | Targeting EI24 | Poor OS | [35][36] |
| miR-139-5p | Downregulated | Targeting ARF6 | Poor DFS | [35] |

**Table3: MicroRNAs Inhibit TNBC**

| **MicroRNA** | **Change in TNBC** | **Mechanism** | **Biological function** | **Reference** |
| --- | --- | --- | --- | --- |
| **inhibit proliferation and invasion** | | | | |
| miRNA-29c | Downregulated | Targeting TGIF2, CREB5 and AKT3 | Inhibit proliferation | [46] |
| miR-182-5p | Downregulated | Targeting FBXW7 to regulate TLR4/NF-κB pathway | Inhibit proliferation and invasion | [47] |
| miR-125b | Upregulated | Targeting APC to regulate Wnt/β-cateninpathway | Inhibit proliferation and invasion | [48] |
| miR-496 | Downregulated | Targeting Del-1 | Inhibit proliferation and invasion | [49] |
| miR-890 | Downregulated | Targeting CD147 | Inhibit proliferation and invasion Induces apoptosis | [50] |
| miR-1301 | Upregulated | Targeting EZH2 | Inhibit proliferation, invasion and colony formation | [51] |
| **inhibit migration** | | | | |
| miR-149 | Upregulated | Targeting CSF1 | Inhibit metastasis | [52] |
| miR‑574‑5p | Downregulated | Targeting SOX2 and BCL11A | Inhibit proliferation, invasion and EMT | [53] |
| miR-33b | Downregulated | Targeting HMGA2, SALL4 and Twist1 | Inhibit metastasis and CSC | [54] |
| miR-124 | Downregulated | Targeting ZEB2 | Inhibit EMT and metastasis | [55] |
| miR-126-3p | Downregulated | Targeting RGS3 | Inhibit proliferation, migration, invasion, colony formation capacity and angiogenesis | [56] |
| miR-130a | Upregulated | Targeting FOSL1 and ZO-1 | Inhibit metastasis and invasion | [57] |
| miR-145 | Downregulated | Targeting ARF6 | Inhibit metastasis and invasion | [58] |

**Table4: Common chemotherapeutic drugs for TNBC**

| **Chemotherapy drugs** | **Mechanism** | **Common Drugs** | **MiRNAs associated with drug resistance** | **Reference** |
| --- | --- | --- | --- | --- |
| Anthracyclines | Acting via DNA insertion, oxidative stress production, and topoisomerase II poisoning | Doxorubicin，  Epidoxorubicin | miR-129-5p，miR-145，  miR-154，miR-181b-2-3p | [67][68][69][70][71][86] |
| Paclitaxel | Promoting intracellular tubulin polymerization and stabilizes abnormal microtubule structures against depolymerization | Docetaxel，  Nab-paclitaxel | miR-26a-5p， miR-142-3p，miR-200，miRNA-5195-3p | [72][73][74][75] |
| Platinum | Cross-linking with bases on the DNA chain, damaging the structure and function of DNA | Carboplatin，  Cisplatin，  Oxaliplatin | miR-105/93-3p，miR-145-5p，miR-423-5p | [34][76][77] |
| Cyclophosphamide | Undergoing hepatic metabolism and producting aldophosphamide，which decomposes into phosphoramide mustard and acrolein in tumor cells to act cytotoxic effects |  |  | [87] |
| Capecitabine | Entering the body and converting into 5-FU, which is incorporated into RNA in a competitive inhibition manner to interfere with protein synthesis |  |  | [88] |
| Gemcitabine | Its main metabolite incorporated into DNA within the cell and mainly acting on the G1/S phase，and inhibiting nucleotide reductase, leading to a decrease in intracellular deoxyribonucleotide triphosphate, and inhibiting deoxycytidine deaminase to reduce the degradation of intracellular metabolites |  |  | [89] |
